# Emergency care utilization in persons with substance related diagnoses

**DOI:** 10.1016/j.abrep.2024.100573

**Published:** 2024-11-23

**Authors:** Wayne Kepner, Natasia S. Courchesne-Krak, Nora Satybaldiyeva, Rekha Narasimhan, Carla B. Marienfeld

**Affiliations:** a Department of Psychiatry & Behavioral Sciences, Stanford University School of Medicine, 1070 Arastradero Road, Palo Alto, CA 94304, USA; b Department of Psychiatry, University of California, San Diego, 9500 Gilman Drive, C101 La Jolla, CA 92093, USA; c Herbert Wertheim School of Public Health and Human Longevity Science, University of California, San Diego, 9500 Gilman Drive, La Jolla, CA 92093, USA

**Keywords:** Emergency health services, Health disparities, Healthcare utilization, Social determinants, Substance use disorder, Substance-related diagnosis

## Abstract

**Background:**

Substance use is a significant contributor to emergency department (ED) visits. Little is known about ED utilization patterns of individuals with substance related diagnosis (SRD). We used electronic health records (EHR) from a large healthcare system in California to examine ED healthcare utilization and socio-demographic characteristics of individuals with SRDs.

**Methods:**

We used EHR data on all adult patients in our health system from April 2012 through September 2019 to conduct adjusted logistic regression models to determine socio-demographic correlates of SRDs (e.g., use, misuse, dependence) and associations between having an SRD and receiving emergency care.

**Results:**

Among the sample (n = 342,651), the majority were female (55.08 %), Non-Hispanic White (58.10 %), with mean age of 48.26 (SD = 18.10), and there were 18,015 (5.26 %) individuals with an SRD. Patients with an alcohol-related diagnosis had the highest odds of visiting the ED (aOR = 3.75), followed by those with opioid (aOR = 3.57) and stimulant-related diagnoses (aOR = 3.48). Individuals with an SRD were more likely to identify as male, Black/African American, Hispanic/Latinx, have no health insurance, and have a serious mental illness. In the adjusted model, those with an SRD were significantly more likely to have ever received emergency care (aOR 3.72 [95 % CI 3.62–3.84]) than those without an SRD.

**Discussion:**

Our study found an association between having an SRD and utilizing emergency health services. Demographic characteristics suggest disparities exist for those with SRDs around gender, race/ethnicity, insurance status, and mental health. These data can help with screening and targeted responses to prevent or provide emergency care.

## Highlights

• Substance-related diagnoses were associated with higher odds of receiving emergency care.
• Alcohol, opioids, and stimulants were strongly associated with receiving emergency care.
• Factors associated with substance-related diagnoses included age, sex, race/ethnicity, and mental health status.

## 1 Introduction

Individuals with SRDs disproportionately engage with emergency medical services including overdose related events (Courchesne-Krak et al., 2023a, Weiss et al., 2017). After controlling for comorbidities, hospitalization (typically preceded by emergency department (ED) presentation) is more prevalent among people with SRD than those without it (Courchesne et al., 2021, Wu et al., 2016). The International Classification of Diseases, 10th edition (ICD-10) classifies substance-related diagnoses (SRDs) into multiple categories encompassing substance intoxication, withdrawal, use, substance use disorders (SUDs), misuse, and dependence (F10.xx-F19.xx). Between 2014–2018, the annual average prevalence of SUD was 9.4 % among ED visits (9.3 million visits) (Suen et al., 2021). Among individuals with high ED use (3–4 annual visits), people with SRDs account for between 25 %–40 % of visits (Urbanoski et al., 2018).

The burden of SRDs is not uniformly distributed across populations; considerable disparities exist across socio-demographic factors. Research suggests that males, and minoritized populations are overrepresented among those with SRDs (Farahmand et al., 2020). These demographic groups may also utilize emergency services at disproportionate rates and may lack adequate health insurance coverage, which raises questions about healthcare accessibility and effectiveness of primary and preventive care among these groups (Courchesne-Krak et al., 2022). At present, there is considerable room for the improvement of effective health services utilization through the reduction of emergency care use and targeted engagement in SRD focused treatment. Our study seeks to further elucidate these concerning trends through the exploration of sociodemographic correlates of SRDs using a large dataset from electronic health records (EHRs). Through this exploration, we aim to inform tailored, targeted healthcare strategies and interventions that address these disparities, ultimately improving both health outcomes and resource utilization. The result would be an improvement in healthcare outcomes, patient experience, and the quality and appropriateness of care, as well as a reduction in healthcare costs by shifting from more intensive and expensive care to less expensive and more targeted care.

In this study, we aim to examine the engagement of emergency healthcare services among individuals with SRDs and determine the sociodemographic correlates of this population. We hypothesize that people with SRDs will be more likely to utilize emergency services compared to those without an SRD. Second, we hypothesize that disparities will exist around common substance related factors such as sex, race/ethnicity, and insurance status. Results from our study will aid researchers and clinicians to better understand the differences and similarities between these groups through the identification of sociodemographic characteristics. Characterizing patient emergency care usage and demographic profiles can help clinicians, administrators, and policymakers to make better informed decisions about program and policy implementations and resource allocation.

## 2 Material and methods

### 2.1 Sample and data source

The present study was conducted at a large academic health system in Southern California which is comprised of multiple hospitals and outpatient clinics. This healthcare system is a referral center with a large urban hospital and a large suburban hospital with a county-wide network of clinics in urban and suburban areas that also serve many rural residents. Clinical services include inpatient and outpatient visits for primary, mental health, addiction, and other medical specialty care. As such, this healthcare system provides a full range of primary and specialty care. This retrospective observational study utilized de-identified EHR data on all patients 18–90 years of age from April 2012 through September 2019. Patients with ages <18 or >90 were excluded to reduce the risk of identification due to the small number of individuals with and without an SRD in these age ranges. These data were collected from the health center’s biomedical informatics team through a standardized data request process which received Institutional Review Board approval at the University of California, San Diego. Data were provided in a secured Health Insurance Portability and Accountability Act (HIPAA) approved Virtual Research Desktop (VRD; supported by the National Institute of Health, Grant UL1TR001442 of CTSA Funding). Patients in the healthcare system were able to opt out of the information exchange from which we gathered our data through an online or mail-in form.

### 2.2 Measures

#### 2.2.1 Outcomes

Emergency healthcare utilization, the main outcome of this study, was defined by the presence of any ED visit in a patient's medical record during the study period and recorded as a binary variable (yes/no). For the primary independent variable (SRD), those who had at least one clinical encounter with an SRD ICD-10 code -at any point in the study period- were included in the SRD group (Supplemental Table 1). Clinical encounters of all types (e.g. outpatient, inpatient, emergency) were used to determine SRD status. All patients in the SRD group could only be counted once regardless of number of visits or number of SRD diagnoses. Those without an SRD for any clinical encounter during the study period were included in the group without an SRD. The secondary independent variables included substance-specific SRD (yes/no) for: alcohol, opioids, stimulants (i.e., cocaine, methamphetamine), nicotine/tobacco, other/poly substances, sedatives, hallucinogen, inhalants and cannabis. Patients could have multiple SRDs and we did not assess interactions or combinations of SRDs.

#### 2.2.2 Covariates

Covariates included age, race (American Indian/Alaskan Native, Native Hawaiian or Pacific Islander, Asian, Black, White, and other or mixed race), and Hispanic/Latinx ethnicity. Self-reported race (e.g., Black, White) and ethnicity (e.g., Hispanic/Latina, African American, Caucasian) are separate categories in this EHR. Those who selected “Unknown” or chose not to disclose their race/ethnicity were categorized as “Unknown”. Marital status was also assessed and categorized as single, divorced/separated/widowed, married/significant other, or “other”. Health insurance coverage was identified as private [e.g., commercial, managed care], public [e.g., Medicaid, Medicare, including managed plans], or no insurance at the time of the encounter. The Charlson Comorbidity Index was used to create a summary variable that captures medical health severity (≥ 1 Charlson Comorbidity; ICD-10 codes available upon request) (Charlson et al., 1987). A serious mental illness diagnosis (SMI; yes/no) included any ICD-10 code for schizophrenia, schizotypal disorder, persistent delusional disorder, schizoaffective disorder, other psychotic disorder not due to a substance or known physiological condition, unspecified psychosis, manic episode, bipolar disorder, or major depressive symptom severe (ICD-10 codes available upon request). A dichotomous variable was created for SMI (yes/no) in which those who reported one or more SMI were categorized as “yes”. A non-SMI included any ICD-10 code for brief psychotic disorders, major depressive disorder mild or moderate, persistent mood disorder, reaction to severe stress, and/or adjustment disorders (includes post-traumatic stress syndrome), obsessive compulsive disorder, phobic anxiety disorder, other anxiety disorder, eating disorder, specific personality disorder, and/or impulse disorder (ICD-10 codes available upon request).

### 2.3 Statistical analysis

To determine sociodemographic factors associated with having an SRD compared to those without an SRD, we used bivariate analysis in the sample using Chi-squared (X^2^) tests of significance for categorical data. To determine the effect/magnitude of the associations, unadjusted odds ratios (ORs) were calculated and reported. Effect sizes for dichotomous and categorical measures were used in addition to p-values and ORs to identify meaningful differences. However, OR confidence intervals that cross 1 (indicating that there was no significant difference and p-values significant at 0.05) were used to determine whether a covariate would be included in the final multivariable regression. To examine the potential for multicollinearity we conducted a variance inflation factor (VIF) analysis for each independent variable. Two sets of multivariable models were used: (1) the first set of models used SRD as the outcome variable and aimed to identify correlates of SRDs; (2) the second set of models treats ED utilization as the outcome variable to examine how SRDs influence ED utilization. Missing data was minimal (<1%) for all variables; we used a complete case analysis approach and excluded observations with missing data from the regression models. Standardized betas (β), standard errors (SE[β]), adjusted odds ratios (AOR) and the respected confidence intervals and p-values were reported. To determine the effect/magnitude of the association between SRD and the main outcome (ED visits), unadjusted odds ratios (ORs) were calculated and reported. Standardized betas (β), standard errors (SE[β]), adjusted odds ratios (AOR) and the respective confidence intervals and p-values were reported.

## 3 Results

### 3.1 Sample characteristics

There were a total of 18,015 patients with any ICD-10 code for an SRD associated with their visit to the health system from April 2012 through September 2019 (Table 1). The total cohort (n = 342,651) was mostly female (55.08 %), White race (58.10 %), and Non-Hispanic ethnicity (74.80 %) with a mean age of 48.3 (SD = 18.10). The two most frequent SRDs among the patients in the sample were alcohol related diagnosis (38.78 %) and nicotine related diagnosis (31.21 %) (Table 2).

**Table 1:** Demographic characteristics of patients in a major hospital system, stratified by the presence or absence of substance-related diagnoses (SRD).

**Characteristic**	**Total Sample**	**SRD**	**No SRD**	***p* value**
**All** (n [%])	342,651 (100)	18,015 (5.26)	324,636 (94.74)	0<.001
**Sex** (n [%])				0<.001
Male	153,877 (44.91)	11,314 (62.80)	142,563 (43.91)	
Female	188,744 (55.08)	6,696 (37.17)	182,048 (56.08)	
Unknown	30 (0.01)	5 (0.03)	25 (0.01)	
**Age** (range 18–90; M ± SD)	48.26 ± 18.10	46.84 ± 14.91	48.34 ± 18.26	0<.001
**Age Categories** (n [%])				0<.001
18–24 years	35,735 (10.44)	1,421 (7.91)	34,314 (10.58)	
25–39 years	90,391 (26.40)	4,615 (25.70)	85,776 (26.44)	
40–54 years	81,689 (23.90)	5,776 (32.17)	75,913 (23.40)	
55–64 years	61,702 (18.02)	4,126 (22.98)	57,576 (17.75)	
65 + years	72,839 (21.30)	2,019 (11.24)	7,082 (21.83)	
**Race** (n [%])				0<.001
Asian	31,820 (9.29)	596 (3.32)	31,224 (9.63)	
American Indian or Alaska Native	1,490 (0.44)	107 (0.60)	1,383 (0.43)	
Black or African American	18,780 (5.20)	1,910 (10.64)	18,780 (5.49)	
Native Hawaiian or Pacific Islander	1,610 (0.47)	64 (0.36)	1,546 (0.48)	
Other or Mixed Race	71,301 (20.83)	2,866 (15.96)	68,435 (21.10)	
White	198,920 (58.10)	12,029 (66.99)	186,891 (57.61)	
Unknown*	18,435 (5.38)	385 (2.14)	18,050 (5.56)	
**Hispanic/Latinx** (n [%])				0<.001
Yes	69,948 (20.43)	2, 944 (16.39)	67,004 (20.65)	
No	256,003 (74.78)	14,678 (81.74)	241,325 (74.39)	
Unknown*	16,405 (4.79)	335 (1.87)	16,070 (4.95)	
**Marital Status** (n [%])				0<.001
Single	130,457 (38.11)	10,030 (55.86)	120,427 (37.12)	
Married/ significant other	160,081 (46.7)	4,412 (24.57)	155,669 (47.99)	
Divorced, separated, widowed	44,180 (12.90)	3,283 (18.28)	40,897 (12.61)	
Other	1,693 (0.49)	61 (0.34)	1,632 (0.50)	
**Insurance Type** (n [%])				0<.001
Private	194,838 (56.92)	9,063 (50.31)	185,775 (57.28)	
Public	95,400 (27.87)	6,009 (33.36)	89,391 (27.56)	
None	52,078 (15.21)	2,943 (16.34)	49,135 (15.15)	
**Serious Mental Illness** (n [%])	1,935 (0.56)	462 (2.56)	1473 (0.45)	0<.001
**Non-Serious Mental Illness** (n [%])	6,953 (2.08)	1,024 (5.68)	5,929 (1.78)	0<.001
Charlson Comorbidity** (n [%])	32,724 (9.52)	1,834 (10.18)	30,890 (8.99)	0<.001

* Unknown = Patient cannot or refuses to declare race.

** Preexisting health condition defined by any positive value for the Charlson Comorbidity Index.

**Table 2:** Frequency and percentage of substance related diagnosis (SRD) by substance.

Substance Related Diagnosis by Substance***	(n [%])
Alcohol related diagnosis	6,987 (38.78)
Nicotine related diagnosis	5,623 (31.21)
Other/Poly related diagnosis	2,824 (15.68)
Stimulant related diagnosis	2,765 (15.35)
Opioid related diagnosis	2,489 (13.81)
Cannabis related diagnosis	1,045 (5.80)
Sedative related diagnosis	449 (2.49)
Cocaine related diagnosis	336 (1.86)
Hallucinogen related diagnosis	37 (0.21)
Inhalant related diagnosis	10 (0.56)
Total Substance Related Diagnosis***	22,565 (125.26)

*** Patients can have more than one SRD diagnosis which can lead to values summing over 100 %.

### 3.2 Substance-related diagnoses (SRD)

Compared to individuals without an SRD, individuals with an SRD were more likely to identify as male (62.80 % vs 43.91 %), Black or African American (10.64 % vs. 5.49 %), have public health insurance (33.36 % vs. 27.56 %) or no health insurance (16.34 % vs. 15.15 %), and have a serious (2.56 % vs. 0.45 %) and non-serious mental illness (5.68 % vs. 1.78 %). Individuals with an SRD were less likely to identify as Hispanic or Latinx (16.39 % vs. 20.65 %) and be married or have a significant other (24.57 % vs. 47.99 %) (Table 1). The adjusted regression model revealed significant associations with SRDs and several socio-demographic factors (Table 3). Specifically, middle-aged adults, males, and both serious and non-serious mental illnesses were all strongly linked to increased likelihood of SRDs.

**Table 3:** Adjusted regression models for socio-demographic factors associated with any substance related diagnosis (SRD).

**Characteristic**	***B***	**Se**	**aOR**	**95 % CI**	**P value**
**Age Categories**					
18–24 years	Ref.				
25–39 years	0.493	0.050	1.63	1.53–1.74	0<.0001
40–54 years	0.855	0.067	2.35	2.21–2.50	0<.0001
55–64 years	0.777	0.067	2.17	2.03–2.32	0<.0001
65+ years	−0.292	0.037	0.75	0.69–0.81	0<.0001
**Sex**					
Female	Ref.				
Male	0.723	0.025	2.06	2.00–2.13	0<.0001
Unknown^	2.024	0.391	7.58	2.70–21.28	0.002
**Race**					
White	Ref.				
Asian	−1.038	0.082	0.36	0.33–0.39	0<.0001
American Indian or Alaska Native	0.070	0.078	1.07	0.88–1.31	0.520
Black or African American	0.186	0.045	1.20	1.14–1.27	0<.0001
Native Hawaiian or Pacific Islander	−0.491	0.114	0.61	0.47–0.79	0<.0001
Other or Mixed Race	−0.350	0.042	0.70	0.67–0.74	0<.0001
Unknown^	−0.785	0.043	0.46	0.41–0.51	0<.0001
**Hispanic/Latinx**					
No	Ref.				
Yes	−0.232	0.029	0.79	0.75–0.83	0<.0001
Unknown^	−0.675	0.045	0.51	0.45–0.58	0<.0001
**Marital Status**					
Single	Ref.				
Married, living as married, significant other	−1.011	0.084	0.36	0.35–0.38	0<.0001
Divorced, separated, widowed	0.095	0.087	1.10	1.05–1.15	0<.0001
Other	−0.375	0.111	0.69	0.53–0.89	0.003
Unknown	−0.535	0.092	0.59	0.50–0.69	0<.0001
**Insurance Type**					
Private	Ref.				
Public	0.501	0.019	1.65	1.59–1.71	0<.0001
None	0.095	0.024	1.10	1.05–1.15	0.003
**Serious Mental Illness (Yes vs No)**	0.481	0.030	2.62	2.33–2.94	0<.0001
**Non Serious Mental Illness (Yes vs No)**	0.470	0.019	2.54	2.36–2.74	0<.0001
Charlson Comorbidity^**^(Yes vs No)	0.042	0.013	1.09	1.03–1.15	0.002

* Covariates in the adjusted model include: gender, race, ethnicity, age, insurance, marital status, Charlson comorbidity index, serious mental illness, non-serious mental illness and year of service. ** Preexisting health condition defined by any positive value for the Charlson Comorbidity Index.

^ Unknown = Patient cannot or refuses to declare.

### 3.3 Emergency department (ED) visits

**Fig. 1:**
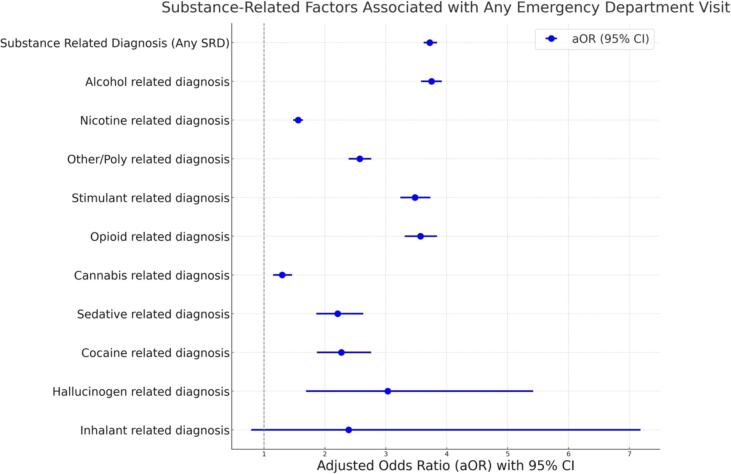
Substance-related factors associated with any emergency department visit.

There was a significant association between having an SRD and receiving emergency care (AOR = 3.72, 95 %CI = 3.62–3.84) after adjustment for covariates. Other than inhalant-related diagnosis (AOR = 2.39, 95 %CI = 0.79–7.18), individuals with all of the different substance specific diagnoses were more likely to have ever visited the emergency department (Table 4) (Fig. 1). Patients with an alcohol-related diagnosis had the highest odds of visiting the emergency department (AOR = 3.75, 95 %CI = 3.58–3.92), followed by those with an opioid-related diagnosis (AOR = 3.57, 95 %CI = 3.31–3.84), and those with a stimulant-related diagnosis (AOR = 3.48, 95 %CI = 3.24–3.73). Although those with a cannabis-related diagnosis had higher odds of emergency department utilization (AOR = 1.30, 95 %CI = 1.15–1.46), this association was weaker than all other substance specific diagnoses. Adjusted regression models for socio-demographics factors associated with an ED visit is presented in Supplemental Table 2.

**Table 4:** Adjusted Regression Models for Substance-Related Factors Associated with Any Emergency Department Visit.

**Characteristic**	***B***	**Se**	**aOR**	**95 % CI**	**P value**
**Substance Related Diagnosis (Any SRD)**	0.658	0.007	3.72	3.62–3.84	0<.0001
Substance Specific Diagnosis**					
Alcohol related diagnosis	0.662	0.113	3.75	3.58–3.92	0<.0001
Nicotine related diagnosis	0.221	0.014	1.56	1.48–1.64	0<.0001
Other/Poly related diagnosis	0.472	0.018	2.57	2.39–2.76	0<.0001
Stimulant related diagnosis	0.623	0.018	3.48	3.24–3.73	0<.0001
Opioid related diagnosis	0.636	0.019	3.57	3.31–3.84	0<.0001
Cannabis related diagnosis	0.128	0.031	1.30	1.15–1.46	0<.0001
Sedative related diagnosis	0.397	0.442	2.21	1.86–2.63	0<.0001
Cocaine related diagnosis	0.410	0.493	2.27	1.87–2.76	0<.0001
Hallucinogen related diagnosis	0.554	0.148	3.03	1.69–5.42	0.0002
Inhalant related diagnosis	0.435	0.281	2.39	0.79–7.18	0.1211

** Covariates in the adjusted model include: all other substances, gender, race, ethnicity, age, insurance, marital status, Charlson comorbidity index, serious mental illness, non-serious mental illness and year of service.

## 4 Discussion

To our knowledge, this is the first study to examine ED visits and socio-demographic characteristics, other diagnoses, and associations among a large sample of individuals with an SRD. First, our analysis revealed a strong association between having an SRD and receiving emergency care which supports our initial hypothesis. These findings are consistent with existing literature which indicates that individuals with SRDs have higher engagement with emergency medical services (Courchesne-Krak et al., 2022, Urbanoski et al., 2018). Also, substance-specific factors (e.g. alcohol, stimulants, opioids) were strongly related to ED visits. We also identified significant sociodemographic correlates of SRDs including age, sex, race/ethnicity, and mental health status. Specifically, our study demonstrated that both serious and non-serious mental health illnesses were associated with over twice the odds of having an SRD. This is consistent with the literature which suggests that individuals with mental health disorders are at a high risk of co-occurring substance use disorders (Jones and McCance-Katz, 2019, Painter et al., 2018). While our study did not assess temporality, these associations are meaningful contributions to our understanding of the multifaceted relationship between SRDs, mental health, and healthcare utilization patterns.

### 4.1 Substance-Specific impacts on ED utilization

For our primary outcome (ED visits), our analysis demonstrated that having any SRD was strongly associated with emergency department visits. Individuals with an SRD had 3.72 times higher odds of having an emergency department visit in their record compared to those without. Specifically, alcohol, stimulant, opioid and other/poly substance-related diagnoses were strongly associated with ED visits. These results demonstrate the complexity of substance use patterns among the population that seek care in emergency settings which indicate the need for providers in emergency settings to be knowledgeable in identifying and treating a variety of SRDs. Further research is warranted to understand the underlying causes and risk factors that lead individuals with SRDs to seek emergency care.

#### 4.1.1 Alcohol-related diagnoses (ARDs)

Our results indicate that alcohol related diagnoses (ARDs) are strongly associated with ED visits. Alcohol related admissions to the ED are complex and can involve both chronic and acute complications (Mirijello et al., 2023, Wolf et al., 2020). Chronic patients often have severe complications that lead them to the ED which might require hospitalization whereas acute cases such as alcohol poisoning can be treated in the ED (Mirijello et al., 2023). These results are concerning considering that alcohol-related ED visits are on the rise in the US; a national study of emergency departments found that the number of acute alcohol-related ED visits increased 51.5 % between 2006 and 2014; the rate increased 40 % in that same period (White et al., 2018). While our study did not differentiate between chronic and acute complications, these data illustrate the challenges associated with caring for individuals with ARD in the ED. Future research should elucidate the specific conditions associated with ARDs that lead to ED visits.

#### 4.1.2 Stimulant-related diagnoses (StRDs)

Our study showed that having a stimulant-related diagnosis (StRDs) was strongly associated with having an ED visit compared to those without StRDs. Studies have shown that patients with SRD are more likely to have high inpatient utilization and higher resource utilization per visit (Howell et al., 2021, Painter et al., 2018). A national survey demonstrated that between 2015–2017, there was a 3-fold increase in methamphetamine use among people who reported past-month heroin use (Strickland et al., 2019). Stimulant use can lead to numerous health issues, including cardiovascular conditions, stroke, hypertensive crises, and severe mental health disorders, all of which can require emergency care. Our findings underscore the importance of evidence-based interventions to reduce the burden on emergency services and improve patient outcomes.

#### 4.1.3 Opioid-related diagnoses (ORDs)

Our study showed that individuals with opioid-related diagnoses (ORDs) had significantly higher odds of having an ED visit compared to those without an ORD. Our findings align with national trends showing an increase in opioid related morbidity and mortality; research suggests that between 2005 and 2015, the national rate of opioid-related emergency department visits increased 99.4 % (Weiss, Elixhauser, et al., 2017). Given the rapid onset of opioid overdose symptoms and the potential for fatal outcomes, patients with ORD are at increased risk of severe health consequences that may require the use of emergency services (Sutter et al., 2017). These data highlight the urgent need for continued focus on tackling the ongoing opioid crisis, including the provision of targeted harm reduction strategies and treatment services. Potential strategies include improving access to naloxone, implementing opioid overdose education programs, expanding medications for opioid use disorder and continued research on overdose prevention services. Comprehensive interventions are urgently needed to reduce the growing trend of opioid-related morbidity and mortality.

### 4.2 Sociodemographic factors and SRDs

#### 4.2.1 Race and ethnicity

Our analysis also revealed significant disparities in SRD prevalence across multiple sociodemographic factors including race/ethnicity; we considered race and ethnicity as proxies for structural factors (e.g., cultural, environmental) rather than as a biological variable. Our findings demonstrated that compared to individuals identifying as White, those identifying as Asians and Native Hawaiians or Pacific Islanders had lower odds for SRD, while those identifying as Blacks or African Americans showed higher odds. Our findings suggest a dissimilar distribution of SRDs in our sample, which is consistent with existing literature that show disparities in substance use and related outcomes across racial/ethnic groups (Courchesne-Krak et al., 2023b, Szaflarski et al., 2011). For example, a recent study demonstrated that compared to White patients, Black and Hispanic patients had higher odds of ED visits involving substance use (Zhang et al., 2021). Another study showed that compared to Whites, individuals identifying as Black or Hispanic had higher rates of alcohol dependence and alcohol-related problems (Mulia et al., 2009). Disparities around race/ethnicity may be driven by complex factors such as healthcare access, economic inequality, structural racism and cultural preferences (Szaflarski et al., 2011). Consequently, future research and intervention programs should prioritize substance-specific training programs for ED physicians and encourage cooperative engagement with communities disproportionately impacted by SRDs. These efforts can potentially reduce the reliance on emergency care, and ultimately decrease healthcare costs and improve health outcomes among vulnerable populations.

#### 4.2.2 Age-related patterns

In our analysis of socio-demographic correlates of SRDs, age was also a significant factor, with all age groups from 25-64 years demonstrating increased odds of an SRD compared to the 18–24 years reference group, with the highest association seen in the 40–54 years group. A reverse trend was observed in individuals 65 years and older, where the odds of an SRD were significantly lower. These data suggest a peak period for SRD related ED care utilization (40–54 years), potentially due to biological factors or social stressors that are prevalent in mid-life, which may decline as individuals age (Compton et al., 2007). However, current research suggests that older adults are engaging in risky substance use at higher rates (Kepner et al., 2023). Future research should explore the reasons and implications of a peak age period of SRD and its associated burdens.

### 4.3 Mental health and SRDs

Our analysis also demonstrated that both serious and non-serious mental illnesses were associated with more than double the odds of an SRD. Individuals with an SMI exhibited an (aOR) of 2.62 which was closely paralleled by those with non-SMI, whose aOR was 2.54. Research suggests that individuals with a past-year mental illness commonly meet diagnostic criteria for a co-occurring past-year SUD; the association is especially strong for serious mental health conditions (Lalli et al., 2021, Priester et al., 2016). High prevalence’s of co-occurrence may be due to overlapping genetic, environmental vulnerabilities and developmental experiences (Asheh et al., 2023, Buckley, 2006). A national study of emergency departments between 2006 and 2013 found large increases among ED visits among those with mental disorders (55.5 % increase for depression, anxiety, or stress reactions and 52 % for psychoses or bipolar disorders) (Weiss, Barrett, et al., 2017). These findings reinforce the urgent need for treatment models that address both mental health and SUDs to improve health outcomes for these patients and decrease reliance on emergency services.

### 4.4 Strengths

Our study has several strengths due to its data source and large sample. We utilized EHR data which allowed us to create and examine a large and complex dataset. Electronic health record data reduces the potential for recall bias which improves the internal validity of our findings, and the robust sample size increases generalizability. By utilizing data from a large sample of patients, we were also able to conduct analyses and provide data on small samples of socio-demographic groups. These sub-group analyses can help identify populations at high risk that may not be captured by large population-based surveys. Finally, the findings of our study related to emergency care use have practical implications for providers and administrators of EDs who can utilize these data for targeted responses and resource allocation.

### 4.5 Limitations

The findings of our study should be interpreted considering inherent limitations to EHR data. First, the reliance on ICD-10 codes to define SRDs might lead to potential misclassification or underestimation of SRDs. We did not analyze specific chief complaints or detailed reasons for visits among patients with SRDs. However, our previous work examined some of these aspects (Courchesne-Krak et al., 2023a). Our ICD-10 codes also did not capture acute poisonings (e.g. T40.0 for opioids) which potentially underestimates the full impact of SRDs. The accuracy and completeness of ICD-10 coding may vary by provider and is also subject to missing data. We used a casewise deletion approach to handle missing data which could bias our findings. Patients who opted out of the healthcare information exchange were not included in our analysis which could limit the generalizability of our findings. Second, the retrospective and observational nature of our study prevents us from drawing any causal inferences, and our analyses may be affected by unmeasured confounders that were not included in the EHR (e.g. education, occupation, social support etc.). Similarly, we did not establish a temporal relationship between SRD and ED utilization. Due to this limitation, future studies should focus on elucidating the temporal order of these associations. Third, our data is from a large healthcare system in Southern California which may limit generalizability. Finally, our study focused on specific SRDs and did not assess interactions which overlook important associations between substances. Small sample sizes for hallucinogen and inhalant related diagnoses may limit interpretations of these associations with ED utilization. Future studies with prospective designs and more rigorous diagnostic and analytical procedures are necessary to validate these findings.

### 4.6 Future research and implications

Future research and interventions should focus on several key areas to mitigate the effects of SRDs in ED. Substance-specific interventions should be developed, particularly targeting alcohol, opioids, and stimulants, which showed the strongest associations with ED visits. Integrated care models addressing both substance use, and mental health issues simultaneously should be further prioritized. Emergency departments should consider implementing screening, brief Intervention, and referral to treatment (SBIRT) programs to identify and assist patients with SRDs (Gardner et al., 2022). Age-targeted programs, especially for the 40–54-year age group, should be considered to address peak periods of SRD prevalence. Older adults, while not normally thought of as substance users, are also increasingly feel the negative results of substance use, and increased training for physicians is warranted for this population (Abu Baker et al., 2024). Culturally sensitive community outreach programs should be created for racial/ethnic groups showing higher odds of SRDs. Finally, research on and implementation of harm reduction strategies, such as safe consumption sites or naloxone distribution programs, should be expanded to reduce the negative health impacts of substance use.

## 5 Conclusion

Our study of patients in a large tertiary healthcare system in Southern California provides insights about emergency care utilization by persons with substance related diagnoses. We found a strong association between having an SRD and ED visits in our sample which highlights the urgent need for targeted interventions and preventative strategies. Specifically, our analysis provided evidence of strong associations between specific SRDs and ED visits. The high prevalence of alcohol, stimulant, opioid and poly-substance use-related diagnoses among patients presenting at the ED reinforces the urgency for healthcare providers in these settings to be trained in identifying and managing the complexities of SRDs. These data describing the burden of SRDs on emergency care services highlights the importance of public health initiatives aimed at reducing the associated harms.

Our study also highlighted important sociodemographic correlates of SRDs, including age, sex, and race, as well as the intersection of SRDs with mental health. These findings reinforce the need for the development of care models that address both mental health and SRDs. Our results revealed substantial disparities in SRD prevalence across sociodemographic variables, which suggests that the burden of SRDs is unequally distributed. Such disparities emphasize the necessity for future research and intervention programs to focus more heavily on communities disproportionately affected by SRDs.

Consequently, our study adds substantial knowledge to the literature by describing emergency healthcare utilization factors and sociodemographic correlates among individuals with SRDs. Future research and interventions should focus on developing substance-specific strategies, particularly for alcohol, opioids, and stimulants, while implementing integrated care models that address both substance use and mental health issues. Emergency departments should consider SBIRT programs, and age-targeted interventions should be implemented, especially for high-risk age groups including older adults. Finally, culturally sensitive community outreach programs and harm reduction strategies should be expanded to better serve diverse populations and mitigate the negative health impacts of substance use.

## Author disclosures

The authors declare that they have no known competing financial interests or personal relationships that could have appeared to influence the work reported in this paper. This research did not receive any specific grant from funding agencies in the public, commercial, or not-for-profit sectors. All authors have approved the final article and agree to be accountable for all aspects of the work.

## Data statement

The dataset used in this study contains sensitive personal health information and therefore cannot be made publicly available due to privacy and ethical considerations. Researchers interested in these data may contact the corresponding author for more information on the access process which may require appropriate institutional review board approval.

## CRediT authorship contribution statement

**Wayne Kepner:** Writing – review & editing, Writing – original draft, Visualization, Methodology, Formal analysis, Data curation, Conceptualization. **Natasia S. Courchesne-Krak:** Writing – review & editing, Writing – original draft, Methodology, Formal analysis, Conceptualization. **Nora Satybaldiyeva:** Writing – review & editing, Writing – original draft, Investigation, Data curation, Conceptualization. **Rekha Narasimhan:** Writing – review & editing, Writing – original draft. **Carla B. Marienfeld:** Writing – review & editing, Writing – original draft, Validation, Supervision, Methodology, Investigation, Data curation, Conceptualization.

## Declaration of competing interest

The authors declare the following financial interests/personal relationships which may be considered as potential competing interests: Wayne Kepner reports financial support was provided by National Institutes of Health under Award Number T32DA035165. Nora Satybaldiyeva reports financial support was provided by National Institutes of Health under Award Number 5T32HL161270-03. If there are other authors, they declare that they have no known competing financial interests or personal relationships that could have appeared to influence the work reported in this paper.

## Data Availability

The authors do not have permission to share data.
