# Supplementary material for: Emergency care utilization in persons with substance related diagnoses
Source: Addict Behav Rep. 2024 Nov 23;20:100573. doi: 10.1016/j.abrep.2024.100573 (PMC11647655; doi:10.1016/j.abrep.2024.100573)
Supplement: Supplementary Data 1 [file mmc1.docx]

| Supplemental Table 1: International Classification of Diseases, Tenth Revision, Clinical Modification (ICD-10) codes for pregnancy related diagnoses, substance-related diagnoses, serious mental illness, and non-serious mental illness | |
| --- | --- |
| Condition/Diagnosis | ICD-10 Codes |
| *Substance-related diagnosis (SRD)* |  |
| Alcohol-related diagnosis | F10.xx |
| Opioid-related diagnosis | F11.xx |
| Cannabis-related diagnosis | F12.xx |
| Sedative, hypnotic, or anxiolytic-related diagnosis | F13.xx |
| Cocaine-related diagnosis | F14.xx |
| Other stimulant-related diagnosis | F15.xx |
| Hallucinogen-related diagnosis | F16.xx |
| Nicotine-related diagnosis | F17.xx |
| Inhalant-related diagnosis | F18.xx |
| Other psychoactive substance-related diagnosis | F19.xx |
| *Serious mental illness (SMI)* |  |
| Schizophrenia | F20.xx |
| Schizotypal disorder | F21.xx |
| Persistent delusional disorder | F22.xx |
| Schizoaffective disorder | F25.xx |
| Manic episode | F30.xx |
| Bipolar disorder | F31.xx |
| Major depressive symptom severe | F32.2-F32.3, F33.2-F33.2 |
| *Non-serious mental illness (Non-SMI)* |  |
| Delusional disorders | F22 |
| Brief psychotic disorders | F23 |
| Other psychotic disorder not due to a substance or known physiologic condition | F28 |
| Unspecified psychosis | F29 |
| Major depressive disorder mild or moderate | F32.0-F32.1, F32.4-F32.9, F33.0-F33.1, F33.4-F33.9 |
| Persistent mood disorder | F39 |
| Reaction to severe stress, and adjustment disorders (includes post-traumatic stress syndrome (PTSD)) | F43.xx |
| Obsessive compulsive disorder | F42.xx |
| Phobic anxiety disorder | F40.xx |
| Other anxiety disorder | F41.xx |
| Eating disorder | F50.xx |
| Specific personality disorder | F60.xx |
| Impulse disorder | F63.xx |

World Health Organization. *ICD-10 : International Statistical Classification of Diseases and Related Health Problems : Tenth Revision, 2nd Ed.*; 2004.
