# Supplementary material for: Emergency care utilization in persons with substance related diagnoses
Source: Addict Behav Rep. 2024 Nov 23;20:100573. doi: 10.1016/j.abrep.2024.100573 (PMC11647655; doi:10.1016/j.abrep.2024.100573)
Supplement: Supplementary Data 2 [file mmc2.docx]

| **Supplemental Table 2. Adjusted Regression Models for Socio-Demographic Factors Associated with Any Emergency Department (ED) Visit** | | | | | |
| --- | --- | --- | --- | --- | --- |
| **Characteristic** | ***B*** | **Se** | **aOR** | **95% CI** | **P value** |
| **Age Categories** |  |  |  |  |  |
| 18-24 years | Ref. |  |  |  |  |
| 25-39 years | -0.0872 | 0.00784 | 0.92 | 0.79 - 0.84 | <.0001 |
| 40-54 years | 0.0364 | 0.00780 | 1.03 | 0.90 - 0.96 | <.0001 |
| 55-64 years | -0.0207 | 0.00891 | 0.98 | 0.85 - 0.90 | 0.02 |
| 65+ years | -0.0361 | 0.00963 | 0.96 | 0.83 - 0.89 | 0.0002 |
| **Sex** |  |  |  |  |  |
| Female | Ref. |  |  |  |  |
| Male | -0.09 | 0.13 | 0.91 | 0.71 - 1.16 | 0.50 |
| Unknown^ | 0.37 | 0.26 | 1.45 | 0.86 - 2.44 | 0.16 |
| **Race** |  |  |  |  |  |
| White | Ref. |  |  |  |  |
| American Indian or Alaska Native | -0.20 | 0.06 | 0.82 | 0.72 - 0.93 | 0.0004 |
| Asian | -0.07 | 0.02 | 0.93 | 0.90 - 0.97 | 0.0003 |
| Black or African American | 0.83 | 0.02 | 2.29 | 2.21 - 2.38 | <.0001 |
| Native Hawaiian or Other Pacific Islander | 0.04 | 0.05 | 1.04 | 0.95 - 1.14 | 0.47 |
| Other or Mixed | 0.11 | 0.02 | 1.12 | 1.07 - 1.17 | <.0001 |
| Unknown^ | -0.76 | 0.03 | 0.47 | 0.44 - 0.50 | <.0001 |
| **Hispanic/Latinx** |  |  |  |  |  |
| No | Ref. |  |  |  |  |
| Yes | 0.57 | 0.02 | 1.77 | 1.71 - 1.83 | <.0001 |
| Unknown^ | -1.00 | 0.03 | 0.37 | 0.35 - 0.39 | <.0001 |
| **Marital Status** |  |  |  |  |  |
| Single | Ref. |  |  |  |  |
| Married, living as married, significant other | 0.07 | 0.03 | 1.07 | 1.01 - 1.14 | 0.003 |
| Divorced, separated, widowed | 0.59 | 0.03 | 1.81 | 1.73 - 1.89 | <.0001 |
| Other | -1.15 | 0.09 | 0.32 | 0.27 - 0.38 | <.0001 |
| Unknown^ | 0.00 | 0.04 | 1.00 | 0.92 - 1.09 | 0.93 |
| **Insurance Type** |  |  |  |  |  |
| Private | Ref. |  |  |  |  |
| Public | -0.08 | 0.01 | 0.92 | 0.90 - 0.94 | <.0001 |
| None | 0.48 | 0.01 | 1.61 | 1.58 - 1.65 | <.0001 |
| **Serious Mental Illness (Yes vs No)** | 0.18 | 0.02 | 1.20 | 1.14 - 1.27 | <.0001 |
| **Non Serious Mental Illness (Yes vs No)** | -0.07 | 0.01 | 0.93 | 0.91 - 0.96 | <.0001 |
| **Charlson Comorbidity** (Yes vs No)** | -0.09 | 0.01 | 0.91 | 0.89 - 0.94 | <.0001 |
| ^Unknown = Patient cannot or refuses to declare  *Covariates in the adjusted model include: gender, race, ethnicity, age, insurance, marital status, Charlson comorbidity index, serious mental illness, non-serious mental illness and year of service | | | | | |
